# Catalase produced by *Candida albicans* protects *Streptococcus mutans* from H_2_O_2_ stress—one more piece in the cross-kingdom synergism puzzle

**DOI:** 10.1128/msphere.00295-23

**Published:** 2023-08-21

**Authors:** Callahan Katrak, Bruna A. Garcia, Louise M. Dornelas-Figueira, Mary Nguyen, Robert B. Williams, Michael C. Lorenz, Jacqueline Abranches

**Affiliations:** 1 Department of Oral Biology, University of Florida College of Dentistry, Gainesville, Florida, USA; 2 Department of Restorative Dental Sciences, University of Florida College of Dentistry, Gainesville, Florida, USA; 3 Department of Microbiology and Molecular Genetics, McGovern Medical School, Houston, Texas, USA; University of Georgia, Athens, Georgia, USA

**Keywords:** *Streptococcus mutans*, *Candida albicans*, biofilms, oral microbiology, oral microbiota, dental plaque, synergism, oxidative stress

## Abstract

Co-infection with *Streptococcus mutans* and *Candida albicans* is associated with dental caries, and their co-cultivation results in enhanced biofilm matrix production that contributes to increased virulence and caries risk. Moreover, the catalase-negative *S. mutans* demonstrates increased oxidative stress tolerance when co-cultivated in biofilms with *C. albicans,* a catalase-producing yeast. Here, we sought to obtain mechanistic insights into the increased H_2_O_2_ tolerance of *S. mutans* when co-cultivated with clinical isolates of *Candida glabrata*, *Candida tropicalis,* and *C. albicans*. Additionally, the *C. albicans* SC5314 laboratory strain, its catalase mutant (SC5314*Δcat1*), and *S. mutans* UA159 and its glucosyltransferase B/C mutant (UA159*ΔgtfB/C*) were grown as single- and dual-species biofilms. Time-kill assays revealed that upon acute H_2_O_2_ challenge, the survival rates of *S. mutans* in dual-species biofilms with the clinical isolates and *C. albicans* SC5314 were greater than when paired with SC5314*Δcat1* or as a single-species biofilm. Importantly, this protection was independent of glucan production through *S. mutans* GtfB/C. Transwell assays and treatment with H_2_O_2_-pre-stimulated *C. albicans* SC5314 supernatant revealed that this protection is contact-dependent. Biofilm stability assays with sublethal H_2_O_2_ or peroxigenic *Streptococcus* A12 challenge resulted in biomass reduction of single-species *S. mutans* UA159 and dual-species with SC5314*Δcat1* biofilms compared to UA159 biofilms co-cultured with *C. albicans* SC5314. *S. mutans* oxidative stress genes were upregulated in single-species biofilms when exposed to H_2_O_2,_ but not when *S. mutans* was co-cultivated with *C. albicans* SC5314. Here, we uncovered a novel, contact-dependent, synergistic interaction in which the catalase of *C. albicans* protects *S. mutans* against H_2_O_2_.

**IMPORTANCE:**

It is well established that co-infection with the gram-positive caries-associated bacterium *Streptococcus mutans* and the yeast pathobiont *Candida albicans* results in aggressive forms of caries in humans and animal models. Together, these microorganisms form robust biofilms through enhanced production of extracellular polysaccharide matrix. Further, co-habitation in a biofilm community appears to enhance these microbes’ tolerance to environmental stressors. Here, we show that catalase produced by *C. albicans* protects *S. mutans* from H_2_O_2_ stress in a biofilm matrix-independent manner. Our findings uncovered a novel synergistic trait between these two microorganisms that could be further exploited for dental caries prevention and control.

## INTRODUCTION

Dental caries is a polymicrobial biofilm-associated disease that is estimated to affect more than billion people worldwide, especially in underserved communities (1,2). The development of dental caries is intimately associated with the high consumption of fermentable carbohydrates like sucrose (table sugar) that favor the growth of acid-producing (acidogenic) and acid-tolerant (aciduric) microorganisms. Among them, *Streptococcus mutans* is considered an important pathogen in dental caries due to its high acidogenicity, aciduricity, and ability to form robust biofilms when sucrose is present (3). *Candida albicans,* a fungal pathobiont, is often co-isolated with *S. mutans* from carious sites in patients with early childhood caries (ECC), dentinal caries, and root caries (4 – 7). Moreover, co-infection with *S. mutans* and *C. albicans* has been associated with recalcitrant oral infections that often result in rampant caries and increased caries reoccurrence in children after clinical interventions (4, 8). In recurring ECC, 80% of children were co-infected with *S. mutans* or *C. albicans,* whereas 68% of the children were co-infected with both species. Importantly, less than 10% of the caries-free children were co-infected with *S. mutans* and *C. albicans* (6). Furthermore, in rats fed a cariogenic diet, co-infection with *S. mutans* and *C. albicans* resulted in more aggressive caries formation than single-species infections (9, 10). This increased virulence was attributed to the upregulation of the glucosyltransferases of *S. mutans* by *C. albicans* and the ability of glucosyltransferase B (GtfB) to bind to *C. albicans* surface, *de facto* enhancing both species’ glucan production (11, 12). Importantly, *C. albicans* possesses several traits that make it a desirable partner for *S. mutans*, including its highly aciduric nature, ability to utilize lactate as a source of energy, secretion of bacterial growth factors, and ability to reduce the oxygen tension in the biofilm (13, 14).

In the oral cavity, hydrogen peroxide (H_2_O_2_) is an important form of reactive oxygen species (ROS) generated as a by-product of microbial metabolism or delivered through oral hygiene products. In fact, H_2_O_2_ has been used intraorally for its antimicrobial properties for over a century (15 – 17). Importantly, net H_2_O_2_ production by commensal oral streptococci is associated with health (18 – 20), as *S. mutans* is susceptible to peroxide stress (3, 21, 22). Previous studies have demonstrated that the ability of commensal streptococci to effectively compete with *S. mutans* is compromised when their H_2_O_2_-producing pathways are disrupted, or following catalase addition, which degrades H_2_O_2_ (18, 23). Moreover, a clinical study showed low salivary H_2_O_2_ levels correlate with increased ECC risk (24). Of note, *C. albicans* is highly H_2_O_2_ tolerant, and while co-cultivation with *S. mutans* improves bacterial survival of H_2_O_2_, fungal survival does not increase (12, 25). *C. albicans* H_2_O_2_ tolerance is primarily dependent on the production of a heme-containing catalase (Cat1), predicted to be localized to multiple intracellular compartments, including the cytosol, mitochondria, and peroxisomes, which is conserved across the genus and whose expression is upregulated in mixed biofilms with *S. mutans* (26 – 28). Contrastingly, *S. mutans* lacks catalase and instead relies on other ROS scavenging systems to cope with fluctuations in ROS levels (29). Thus, we hypothesized that *C. albicans’* catalase confers a competitive advantage to *S. mutans* against peroxigenic streptococci, favoring a dysbiotic cariogenic biofilm. Here, we investigated the role that *C. albicans’* catalase plays in the increased tolerance of *S. mutans* against oxidative stress in dual-species biofilms. We showed that in dual-species biofilms, the catalase produced by *C. albicans* protects *S. mutans* from H_2_O_2_ stress in a contact-dependent manner.

## RESULTS

### Clinical strains of *C. albicans, C. glabrata,* and *C. tropicalis* and the laboratory strain of *C. albicans* SC5314 protect clinical co-isolates of *S. mutans* and the laboratory strain UA159 against acute oxidative stress in dual-species biofilms

**Fig 1:**
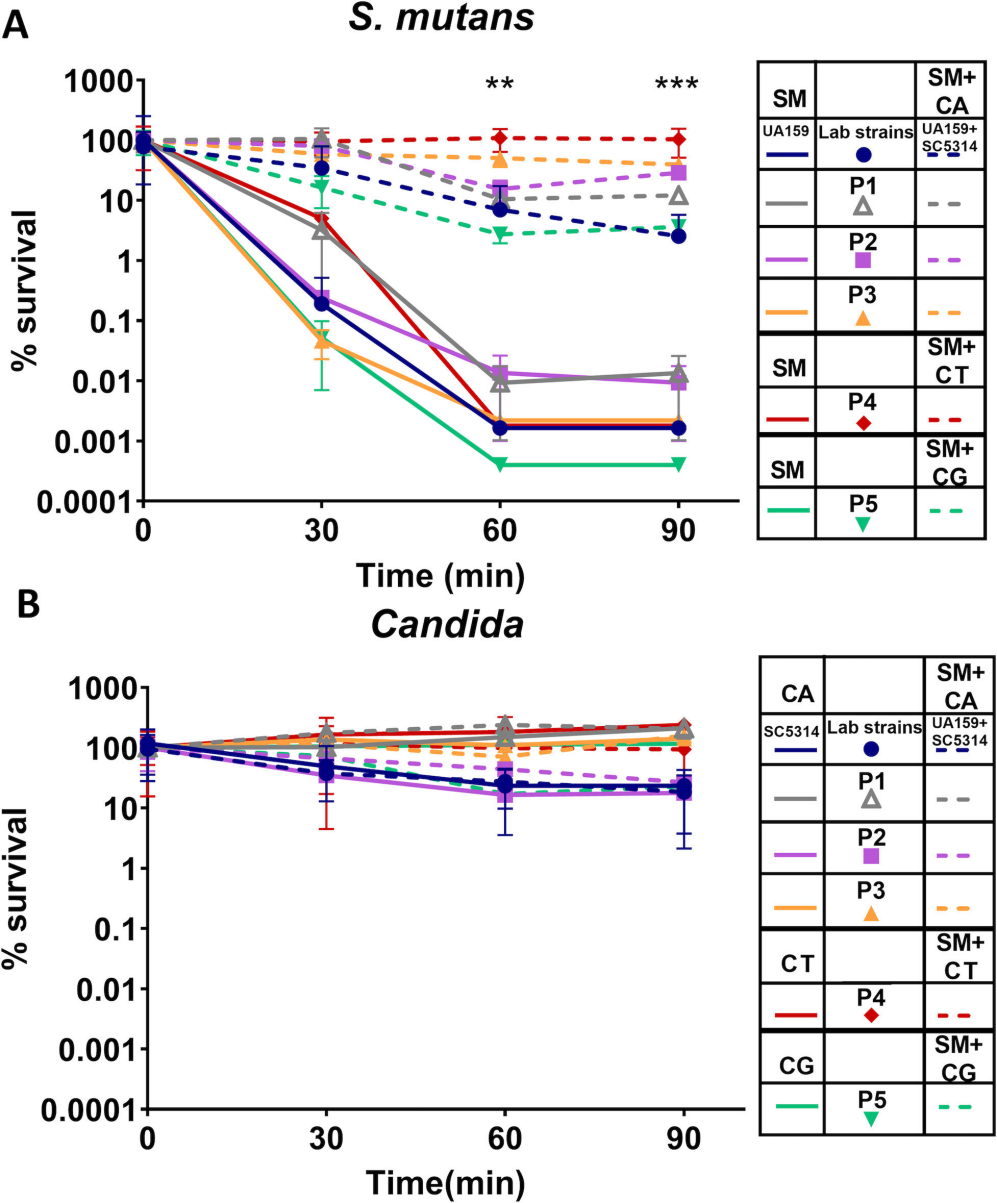
*Candida albicans, C. glabrata*, and *C. tropicalis* protect *S. mutans* against acute oxidative stress in dual-species biofilms. Peroxide tolerance of (**A**) clinical and laboratory strains of *S. mutans* and (**B**) clinical and laboratory strains of *Candida* in 48-h-old single- or dual-species biofilms after exposure to 0.25% H_2_O_2_. The percentage of survival among P1 through P5 represents co-isolated *S. mutans* (SM) and *Candida* (CA-*C. albicans*, *CT-C. tropicalis*, and CG-*C. glabrata*) strains from five distinct ECC patients. P1, P2, and P3 were co-infected with *S. mutans* and *C. albicans*. P4 was co-infected with *S. mutans* and *C. tropicalis*. P5 was co-infected with *S. mutans* and *C. glabrata*. Lab strains UA159: *S. mutans* UA159 and SC5314: *C. albicans* SC5314. Data represent the average and standard deviation of survival percentage compared to CFUs at time 0. Statistical difference between *S. mutans* survival in dual-species biofilm compared to single-species biofilm. **P < 0.01 and ***P < 0.001 (*t*-tests for each single-species vs. dual-species of the same patient).

Here, we explored whether the reported oxidative stress protection of *S. mutans* by *C. albicans* is shared among clinical strains of human-associated *Candida* species and *S. mutans* (12). For this, we utilized a panel of paired *S. mutans* and *Candida* co-isolates from plaque samples of ECC patients, including three co-isolates of *S. mutans* and *C. albicans* (SMP1 & CAP1, SMP2 & CAP2, and SMP3 & CAP3), one co-isolate of *S. mutans* and *C. tropicalis* (SMP4 & CTP4), and one co-isolate of *S. mutans* and *C. glabrata* (SM P5 & CG5) (Fig. 1). Biofilms were exposed to 0.25% H_2_O_2_ (73.5 mM) for up to 90 min and plated for viable CFU determination. In all cases, *S. mutans* clinical isolates demonstrated dramatically enhanced survival in dual-species biofilms with their *Candida* clinical co-isolates compared to single-species biofilms (*P* < 0.001) (Fig. 1A). Also, for *Candida* clinical isolates, compared to single-species biofilms, no changes in survival were observed when they were in dual-species biofilms with *S. mutans* (Fig. 1B). When the lab strains of *C. albicans* SC5314 and *S. mutans* UA159 were co-cultivated in biofilms, we confirmed the previous finding that UA159 had enhanced survival when grown as dual-species biofilms with SC5314 as compared to single-species biofilms (*P* < 0.001) (12, 25). However, SC5314 survival does not appear to benefit from UA159 (Fig. 1).

### *C. albicans’* catalase protects *S. mutans* against acute oxidative stress in dual-species biofilms

After verifying that *Candida*’s protection of *S. mutans* is a common trait among human-associated species and is strain-independent, we continued our studies with the lab strains of *S. mutans* (UA159) and *C. albicans* (SC5314), as mutants of our genes of interest were available. First, we utilized a *C. albicans* catalase mutant (SC5314*Δcat1*) and an *S. mutans* glucosyltransferase B/C-deficient strain (UA159∆*gtfB/C*) to determine whether inactivation of these genes impacts their ability to form biofilms. Dual-species biofilms of UA159 with either *C. albicans* strain (SC5314 or SC5314*Δcat1*) showed increased biomass in relation to UA159 single-species biofilms (*P* < 0.01) (Fig. S1). As expected, the UA159∆*gtfB/C* mutant, a strain with impaired ability to produce extracellular polysaccharide (EPS) matrix, formed poor biofilms when compared to the parent strain UA159 in single-species biofilms (*P* < 0.001). Also, no enhancement of biofilm biomass was observed when UA159∆*gtfB/C* was co-cultivated with SC5314 or SC5314*Δcat1* (*P* > 0.05). Of note, when compared to *C. albicans* SC5314, the *C. albicans* catalase-mutant strain SC5314*Δcat1* showed no impairment in biofilm formation (Fig. S1).

**Fig 2:**
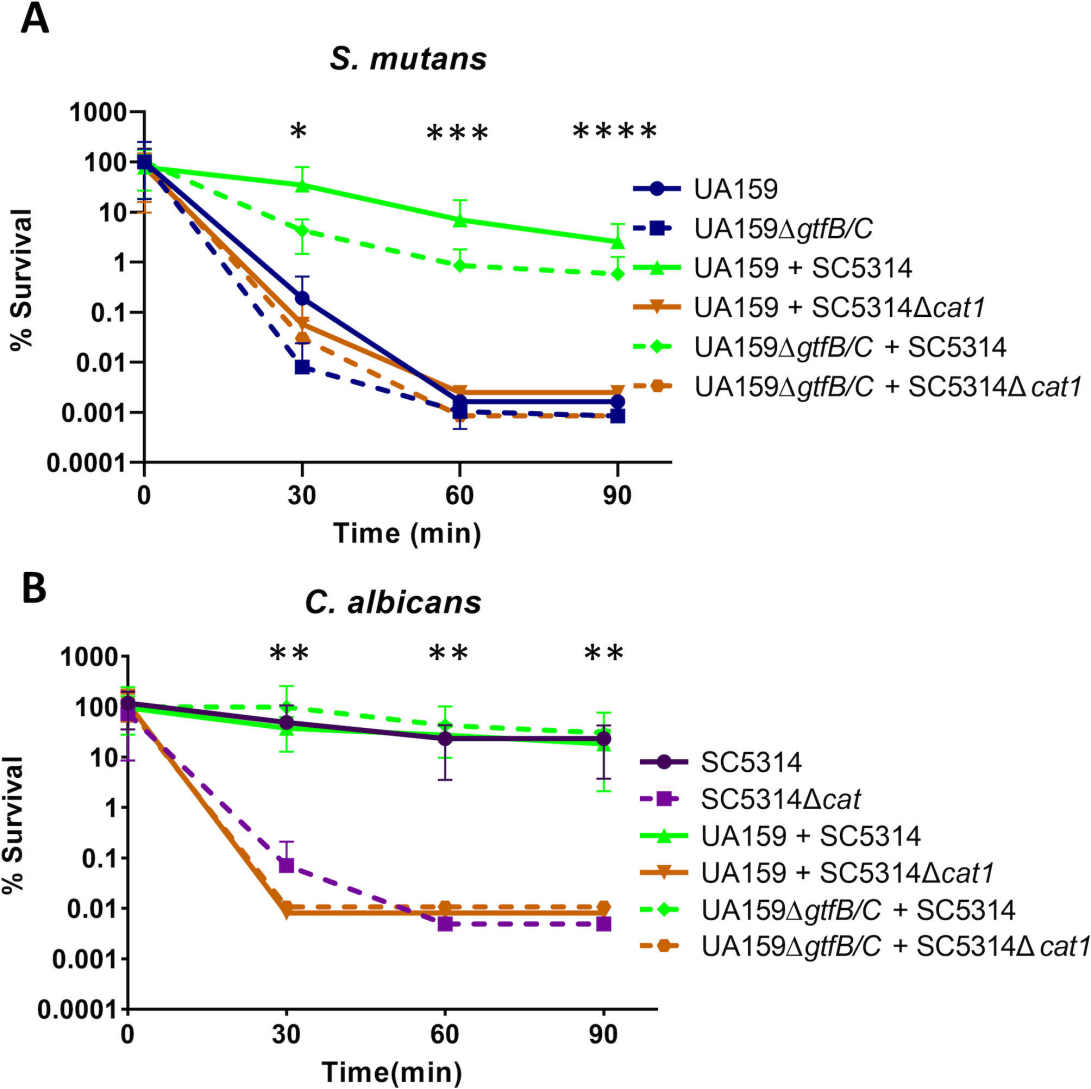
*C. albicans* protects *S. mutans* against acute oxidative stress in dual-species biofilms in a catalase-dependent manner. Peroxide tolerance of (**A**) *S. mutans* and (**B**) *C. albicans* in 48-h-old single- or dual-species biofilms after exposure to 0.25% H_2_O_2_. Data represent the average and standard deviation of survival percentage compared to CFUs at time 0. Statistical difference between survival in dual-species biofilm with *C. albicans* SC5314 versus single-species biofilm or in dual-species biofilm with SC5314*Δcat1* at a specific exposure time is noted with *P < 0.05, ***P < 0.001, and ****P < 0.0001 (one-way ANOVA, followed by Dunn’s multiple comparison tests). Statistical difference between survival in biofilm with *C. albicans* SC5314 versus biofilm with SC5314*Δcat1* at a specific exposure time is noted with **P < 0.01 (one-way ANOVA, followed by Dunn’s multiple comparison tests). UA159: *S. mutans* UA159*,* UA159*ΔgtfB/C: S. mutans* UA159 with glycosyltransferase B/C deletion mutant*,* SC5314: *C. albicans* SC5314*,* and SC5314*Δcat1: C. albicans* SC5314 with catalase deletion mutant.

After determining that inactivation of *C. albicans’* catalase did not affect biofilm biomass in either single-species or dual-species biofilms with *S. mutans*, mature single- and dual-species biofilms were exposed to 0.25% H_2_O_2_ for up to 90 min and plated for CFU estimation. We found that the UA159*ΔgtfB/C* strain behaved similarly to UA159 in mono-species biofilms when challenged with H_2_O_2_ (*P* > 0.95) (Fig. 2). *S. mutans* strains (UA159 and UA159*ΔgtfB/C*) were afforded significant protection when grown as dual-species biofilms with SC5314 as compared to single-species biofilms (*P* < 0.0001). Further, UA159-SC5314 and UA159Δ*gtfB/C*-SC5314 biofilms did not differ from each other upon H_2_O_2_ challenge (*P* > 0.95) (Fig. 2A), indicating that robust EPS matrix production does not protect *S. mutans* from lethal doses of H_2_O_2_. However, *C. albicans* SC5314*Δcat1* failed to provide this same protective effect, indicating that *C. albicans’* catalase is a major player in the protection of *S. mutans* against H_2_O_2_. While *C. albicans* SC5314 displayed a high tolerance to H_2_O_2_ that was not impacted by co-cultivation with *S. mutans* (Fig. 2B), the SC5314*Δcat1* mutant was always highly sensitive to H_2_O_2_ when compared to the parent SC5314 strain (Fig. 2B) (*P* < 0.01), as previously reported (30, 31). To confirm that the *C. albicans* catalase mutant did not have other mutations that could affect its tolerance to H_2_O_2_ stress, a second catalase mutant was independently constructed (SC5314*Δcat1#2*) and used to demonstrate that the phenotypes observed are solely due to the lack of a functional catalase (Fig. S2).

### *S. mutans* oxidative stress tolerance is contact-dependent

**Fig 3:**
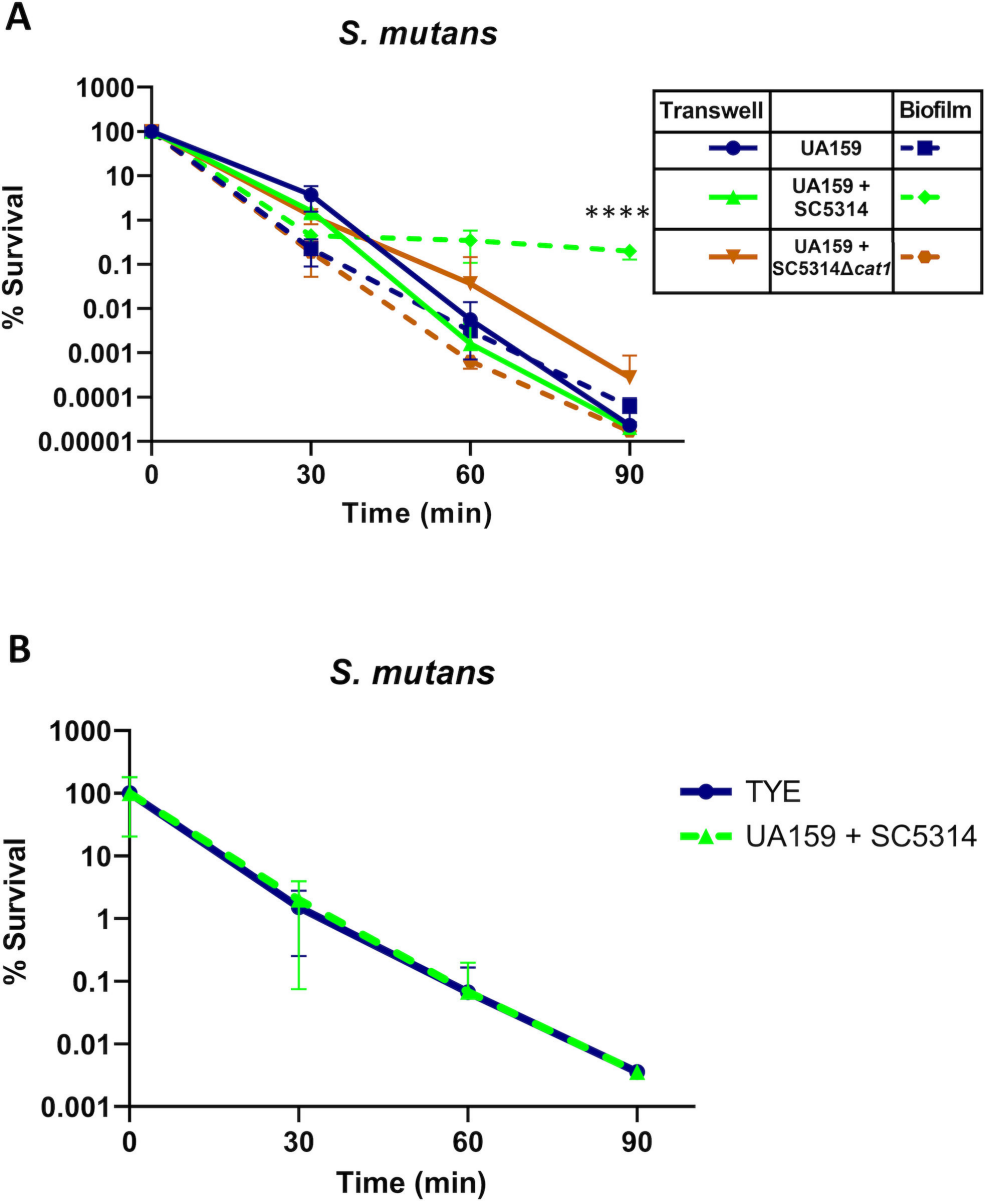
Acute oxidative stress tolerance of *S. mutans* is dependent on contact with *C. albicans*. (**A**) Twelve-hour-old *S. mutans* UA159 biofilms were grown physically separated from *C. albicans* SC5314, SC5314Δ*cat1*, or media only (control) in a transwell apparatus and then challenged for 0, 30, 60, and 90 min with 0.2% (58.8 mM) H_2_O_2_. Single- and dual-species biofilms were grown for 12 h in the bottom well as controls. (**B**) Forty-eight-hour-old biofilms were placed in filter-sterilized supernatant of *C. albicans* SC5314 pre-exposed to H_2_O_2_ or fresh TYE media and were then exposed to 0.25% H_2_O_2_. (A and B) Data represent the average and standard deviation of at least three independent biological replicates and are presented as percent survival compared to CFUs at time 0. Only a statistical difference in survival in dual-species biofilm with SC5314 versus single-species biofilm or in dual-species biofilm with SC5314*Δcat1* or any transwell combination was noted (*P* < 0.0001, one-way ANOVA, followed by Dunn’s multiple comparison tests).

Though *C. albicans’* catalase is predicted to be an intracellular protein, it was experimentally identified on the cell wall and thus is likely secreted (32). We then examined whether the ability of *C. albicans* to protect *S. mutans* is contact-dependent by performing H_2_O_2_ survival assays using a Transwell system that allows diffusion of nutrients and metabolic products while keeping the organisms physically separated. Since 48 h of biofilm development allowed *C. albicans* to penetrate the microporous membrane, a 12-h time point was utilized, and a group of unseparated biofilms was used as a control. Our findings revealed that when physically separated, *C. albicans* cannot protect *S. mutans* from the H_2_O_2_ challenge (Fig. 3A). Further, since it is known that *C. albicans’* catalase expression is stimulated by H_2_O_2_ (27), *S. mutans* survival following exposure to acute oxidative stress was monitored in supernatants obtained from *C. albicans* pre-stimulated with H_2_O_2_. No enhanced survival of *S. mutans* was observed when it was challenged with H_2_O_2_ in the presence of *C. albicans* H_2_O_2_ pre-stimulated supernatant (Fig. 3B). Longitudinally, all groups in the Transwell apparatus and pre-exposed *C. albicans* supernatant presented statistical differences in surviving *S. mutans* CFUs after 60 and 90 min compared to 0 min (*P* < 0.05). All *S. mutans* groups in the Transwell demonstrated reduced survival rates compared to UA159-SC5314 dual-species control biofilms after 90 min (*P* < 0.0001), suggesting that any extracellular catalase is likely bound to the fungal cell wall and not available to protect *S. mutans* at a distance.

### Confocal laser scanning microscopy (CLSM) illustrates the importance of *C. albicans’* catalase in the survival of *S. mutans* during exposure to H_2_O_2_

**Fig 4:**
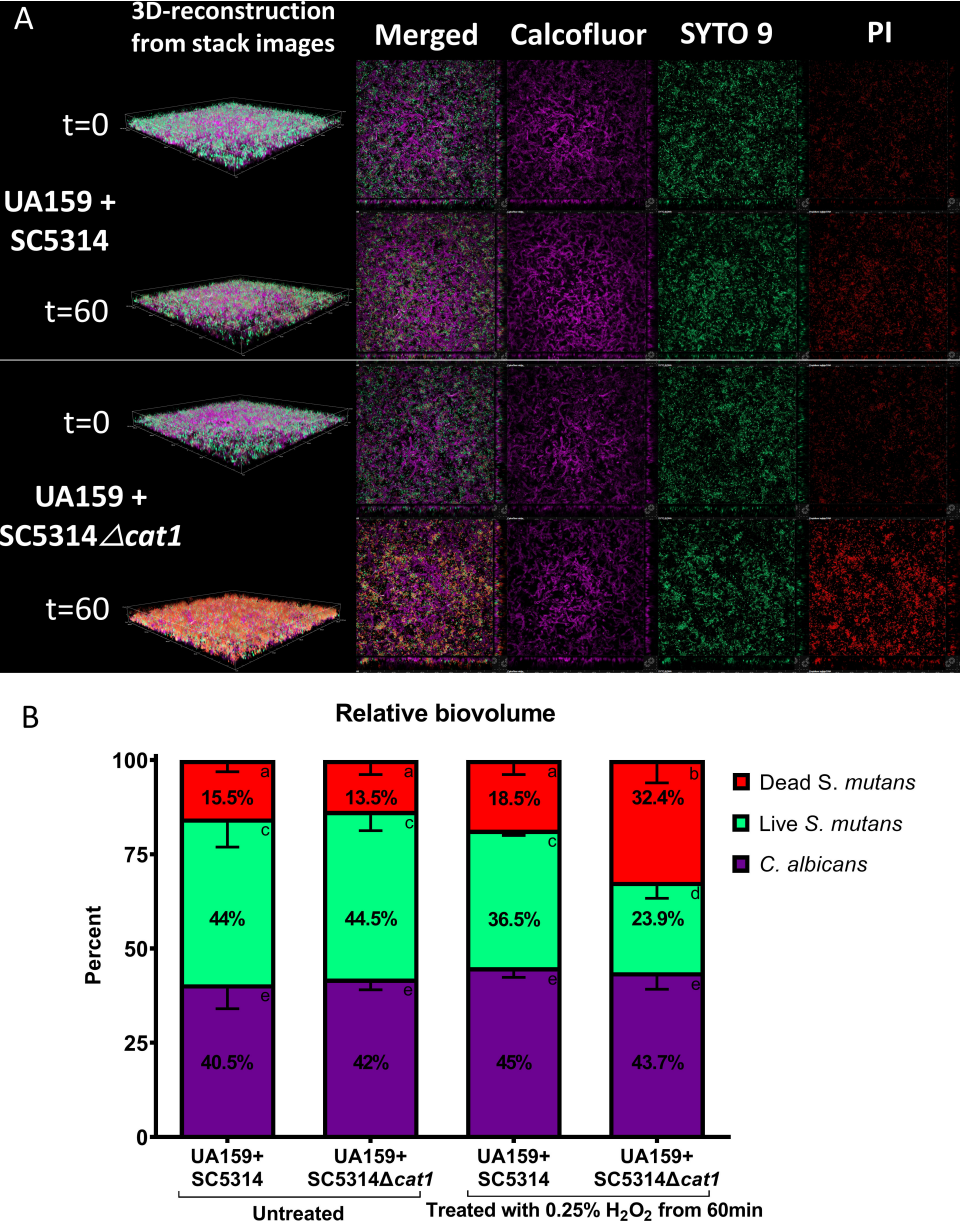
Representative confocal microscopy images and quantification of 48-h-old *S. mutans* and *C. albicans* dual-species biofilms illustrate the role of *C. albicans*’ catalase in the protection of *S. mutans* against H_2_O_2_. The green, red, and purple colors represent live cells of *S. mutans* (SYTO9-: green), dead cells of *S. mutans* (propidium iodide (PI)-red), and all cells of *C. albicans* (Calcofluor-: purple), respectively. The merged 3D reconstruction is presented in the first column, while the other columns comprise a representative Z-layer for merged and individual channels. Biovolume was quantified for each individual component and then normalized by the total components in the sample. (**B**) The proportion of the biovolume from each component for treated and untreated biofilms is shown. Statistical difference between the same component shown: a versus b and c versus d, *P* < 0.01 (one-way ANOVA, followed by Dunn’s multiple comparison tests).

To gain insight into the potential contribution of *C. albicans’* catalase to biofilm architecture, SC5314 and SC5314*∆cat1* were grown as 48 h single- and dual-species biofilms alongside UA159 and analyzed by CLSM. The biofilms were labeled with bacterial live-dead staining (SYTO 9 and propidium iodide-PI) to allow differentiation of live and dead *S. mutans* cells and calcofluor to visualize total *C. albicans* cells. Fig. 4A shows a representative image of a dual-species biofilm with a homogenous distribution of total *C. albicans* cells (live and dead: purple) surrounded by *S. mutans* (live: green, and dead: red). In the untreated group, there was no difference in biofilm architecture between UA159-SC5314 and UA159*-*SC5314*Δcat1* dual-species biofilms (Fig. 4A). Also, SC5314 and SC5314*Δcat1* single-species biofilms labeled with FUN1 (live cells) and Calcofluor (all cells) were comparable (Fig. S2), indicating that catalase production does not contribute to the inherent biofilm architecture of *C. albicans*. Of note, contrary to previous studies showing differences in hyphal formation (33, 34), we did not observe any difference in hyphal abundance between SC5314 and SC5314*∆cat1* (Fig. S3A).

Next, the dual-species biofilms were treated with 0.25% H_2_O_2_ for 60 min and stained. While the untreated UA159-SC5314 and UA159-SC5314*Δcat1* biofilms displayed no significant differences in the quantification of live or dead *S. mutans* or *C. albicans* cells (*P* > 0.1) (Fig. 4), the UA159-SC5314∆*cat1* biofilms treated with H_2_O_2_ displayed a higher abundance of dead *S. mutans* cells throughout the biofilm when compared to UA159-SC5314 biofilms (*P* < 0.01) (Fig. 4B). Representative images of H_2_O_2_-treated and untreated single-species *S. mutans* and *C. albicans* (SC5314 and SC5314*∆cat1*) biofilms are shown in Fig. S3.

### *C. albicans* restores *S. mutans* biofilm formation in the presence of a sublethal dose of H_2_O_2_ or the peroxigenic commensal *Streptococcus* A12

**Fig 5:**
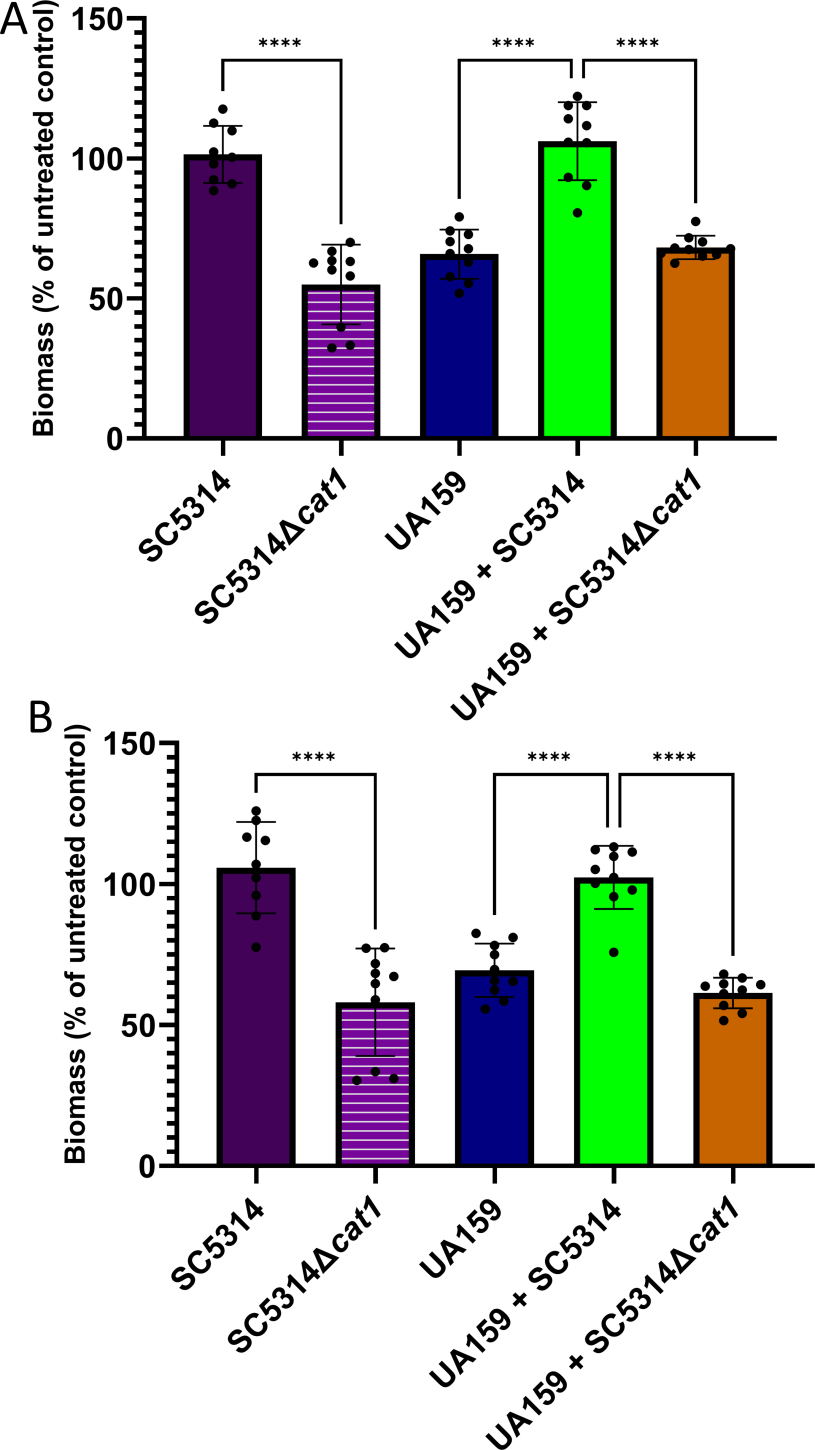
*C. albicans*’ catalase activity promotes biofilm growth and accumulation during challenge with 0.005% H_2_O_2_ or the peroxigenic *Streptococcus* A12 either as a single- or dual-species biofilm with *S. mutans.* Eight-hour-old biofilms were treated with 0.005% H_2_O_2_ (A), *Streptococcus* A12 (B), or left untreated, and biofilms were allowed to grow for an additional 12 h. Crystal violet staining of the 20 h biofilms was normalized to untreated wells. Data represent the average and standard deviation of (OD_575_ for treated wells)/(OD_575_ for untreated wells). Statistical difference: ****P < 0.0001 (one-way ANOVA, followed by Sidak’s multiple comparison tests).

After investigating the ability of *C. albicans* to protect *S. mutans* from a lethal dose of H_2_O_2_ in mature biofilms, we examined whether exposure to sublethal oxidative stress interferes with the growth and accumulation (stability) of early biofilms of UA159 and *C. albicans* (SC5314 and SC5314Δ*cat1*). Here, we utilized either 0.005% H_2_O_2_ (1.446 mM; Fig. 5A), a concentration that is estimated to be produced by peroxigenic streptococci (18), or direct inoculation with *Streptococcus* A12, a peroxigenic oral *Streptococcus* associated with health (Fig. 5B) (22). While 0.005% H_2_O_2_ does not affect *S. mutans* viability (data not shown), oxidative stress can impact *S. mutans* behavior even below inhibitory concentrations (35). Upon exposure to 0.005% H_2_O_2_ or *Streptococcus* A12*,* UA159 single-species biofilms and UA159-SC5314∆*cat1* dual-species biofilms were less stable than UA159-SC5314 dual-species biofilms (*P* > 0.0001). As for *C. albicans* (Fig. 5), SC5314*Δcat1* single-species biofilm stability was reduced compared to SC5314 single-species biofilms a following 0.005% H_2_O_2_ or *Streptococcus* A12 challenge. To rule out other antagonistic mechanisms employed by *Streptococcus* A12 that were not interfering with the biofilms, we also exogenously added catalase at the time of *Streptococcus* A12 inoculations (Fig. S4). We found that the addition of exogenous catalase protected these biofilms against the inhibitory effect of *Streptococcus* A12 (*P* < 0.05). Our findings suggest that upon exposure to sublethal H_2_O_2,_ including H_2_O_2_ produced by *Streptococcus* A12, *C. albicans’* catalase confers stability to both *C. albicans* mono-species biofilms and to dual-species biofilms with *S. mutans*.

### *C. albicans* protects *S. mutans* from sensing oxidative stress

**Fig 6:**
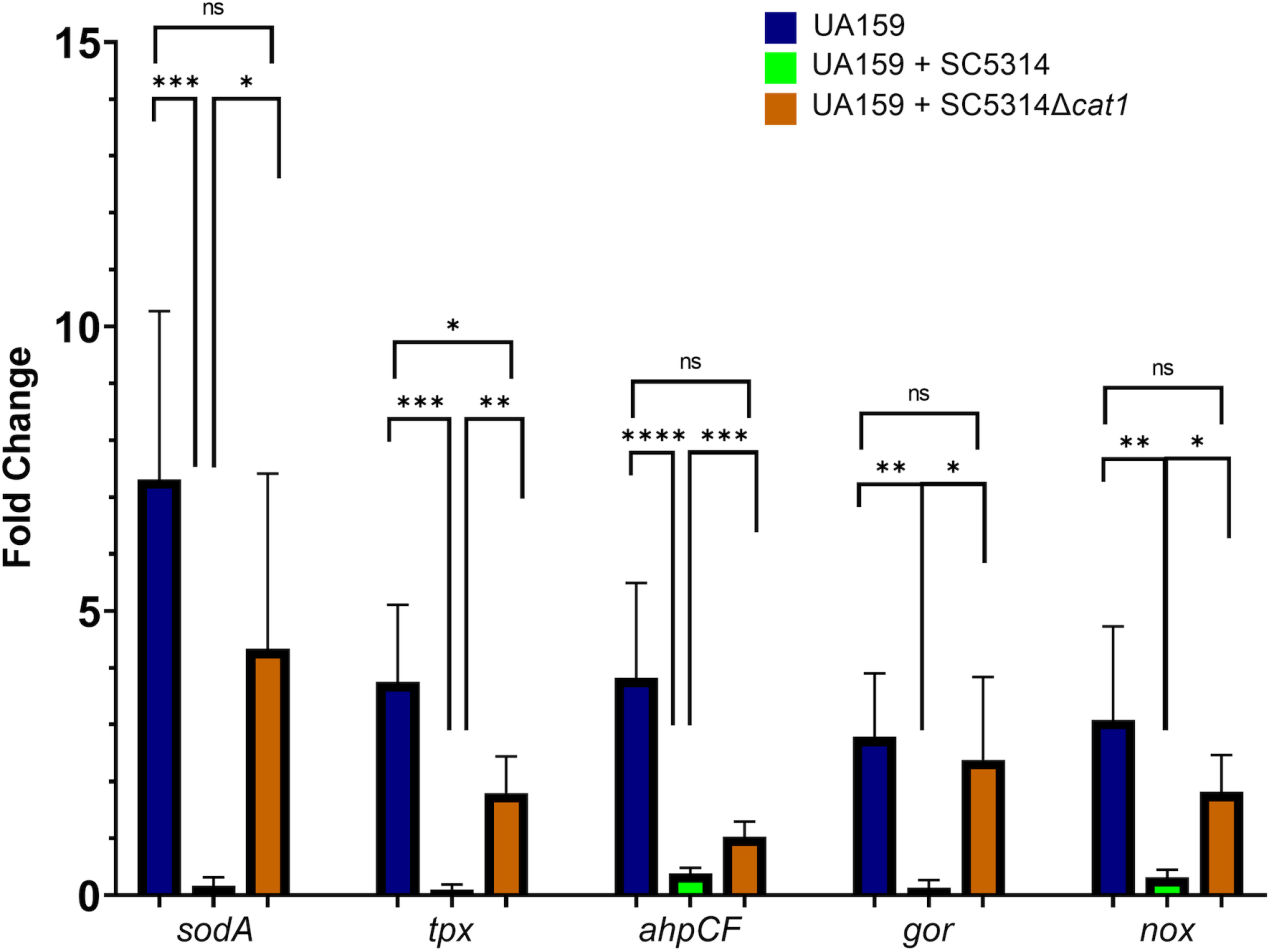
Expression of *S. mutans* oxidative stress genes was not induced after H_2_O_2_ exposure in dual-species biofilms *with C. albicans* SC5314. Twenty-four-hour-old *S. mutans* UA159 alone or in combination with SC5314 or SC5314*Δcat1* biofilms were exposed to 0.005% H_2_O_2_ for 5 min. Then, the biofilms were harvested, and gene expression was assessed by qRT-PCR. Data were normalized by time 0 (biofilms not exposed to H_2_O_2_) and represented the average and standard deviation of fold change. Statistical differences were found between groups with *P < 0.05, **P < 0.01, ***P < 0.001, and ****P < 0.0001 (ANOVA followed by Dunn’s).

When experiencing oxidative stress, *S. mutans* upregulates stress response pathways to cope with the damaging effects of ROS (29, 36, 37). To gain insight into the *S. mutans’* oxidative response during growth in dual-species biofilms with *C. albicans*, we quantified the expression of selected oxidative stress genes of UA159 grown in single- or dual-species biofilms with either SC5314 or SC5314*∆cat1*, with or without exposure to 0.005% H_2_O_2_. The *S. mutans* oxidative stress genes investigated were superoxide dismutase (*sodA*)*,* thiol peroxidase (*tpx*)*,* alkyl hydroperoxide (*ahpCF*)*,* glutathione reductase (*gor*)*,* and H_2_O-forming NADH oxidase (*nox*), which are shown to be upregulated upon peroxide stress (35). To ensure our *S. mutans* primers would not amplify *C. albicans* genes, we blasted our primers against the genome of *C. albicans* SC5314 and no alignment was observed (data not shown). When compared to untreated UA159 single-species biofilms, the expression of all genes was upregulated at least threefold in H_2_O_2_-treated single-species UA159 biofilms, but no induction of these genes was seen in UA159-SC5314 biofilms (*P* < 0.01) (Fig. 6). All five genes were found to be upregulated in dual-species biofilms of UA159-SC5314*∆cat1* as compared to those with UA159-SC5314 (*P* < 0.01). When comparing dual-species biofilms of UA159-SC5314*∆cat1* to UA159 single-species biofilms, the induction of the *tpx* gene was smaller in UA159-SC5314*∆cat1* than that observed for UA159 only (*P* < 0.01). Although not statistically significant, we observed a similar trend of decreased induction of *ahpCF*, *sodA*, *gor,* and *nox* in UA159-SC5314*∆cat1* compared to UA159 single-species biofilms (Fig. 6).

## DISCUSSION

Clinical, *in vivo,* and *in vitro* studies have shown that the cross-kingdom synergistic relationship between *S. mutans* and *C. albicans* increases the persistence of these organisms in the oral cavity and results in enhanced cariogenicity (4, 5, 8, 10, 12, 38, 39). However, most studies evaluating synergistic mechanisms between these microorganisms have centered on the increased production of EPS matrix by *S. mutans* in dual-species biofilms (12, 40 – 43). Moreover, since *S. mutans* is highly susceptible to ROS, it benefits from the presence of *C. albicans* in dual-species biofilms by displaying increased hydrogen peroxide stress tolerance (12). Here, by using clinical co-isolates of *S. mutans* and *C. albicans*, *C. glabrata,* and *C. tropicalis*, we showed that the protection of *S. mutans* against H_2_O_2_ by human-associated *Candida* species is not restricted to laboratory strains or *C. albicans*. Then, using an *S. mutans* strain with impaired ability to produce EPS matrix and a catalase-deficient *C. albicans* mutant strain, we showed that the increased oxidative stress tolerance of *S. mutans* in dual-species biofilms with *C. albicans* is independent of EPS matrix production but dependent upon the catalase produced by *C. albicans*. Thus, the present study expanded, beyond the EPS matrix, the mutualistic repertoire of *S. mutans* and *C. albicans*. In addition, our findings point to *S. mutans* also benefiting from the presence of other human-associated *Candida* species.

Biofilm matrices of cross-kingdom biofilms are often more robust, as seen with dual-species biofilms of *C. albicans* and multiple bacteria, including *Enterococcus faecalis*, *Staphylococcus epidermidis, S. aureus,* and *S. mutans* (11, 44 – 46). These robust matrices create diffusion barriers against environmental stressors. For example, *C. albicans* has increased resistance to fluconazole in biofilms with *S. epidermis* or *S. aureus* (44, 45). Moreover, the increased matrix production in *S. mutans-C. albicans* biofilms appears to serve as a diffusion barrier, protecting both species from the antimicrobial activity of chlorhexidine (12). In *S. mutans-C. albicans* biofilms, the increased EPS-matrix production results from *C. albicans* presence, causing upregulation of *S. mutans’* glucosyltransferase genes (*gtfB*, *C*, and *D*) (10). GtfB produces insoluble glucans, and GtfC produces both soluble and insoluble glucans, contributing to the bulk of the EPS matrix in the presence of sucrose. Of note, *S. mutans* GtfD produces soluble glucans that are thought to serve mostly as a nutrient reservoir. Hence, while our *S. mutans* UA159*ΔgtfB/C* strain has intact *gtfD*, the soluble glucans produced by GtfD represent a small fraction of the EPS produced in *S. mutans* biofilms (47). Importantly, *in vitro* studies revealed that planktonic cultures and mature biofilms of *S. mutans* are equally susceptible to H_2_O_2_ challenge (48), likely because the diffusion properties of H_2_O_2_ and H_2_O are similar (49). Our study confirms the increased acute H_2_O_2_ tolerance previously seen for S. *mutans* in dual-species biofilms with *C. albicans* (12); however, our experiments using an EPS-deficient *S. mutans* mutant strain showed that this survival enhancement is independent of the EPS matrix.

Importantly, *C. albicans* protection of bacterial species from oxidative stress is not limited to *S. mutans.* When in biofilms, *C. albicans* protected the anaerobic species *Clostridium perfringens* and *Bacteroides fragilis* from the stress of an aerobic environment (50). Additionally, *C. albicans* has been shown to modulate the early oral bacterial microbiome by increasing the abundance of facultative and strict anaerobes (51). The mechanism involved in this protection conferred by *C. albicans* to less aerotolerant species is uncharacterized, but we speculate that consumption of O_2_ by respiration and the production of ROS detoxifying enzymes like catalase and the extracellular superoxide dismutases (Sod4,5,6) by *C. albicans* promote the survival of more oxygen-sensitive species. Here, by using a catalase-deficient strain of *C. albicans* SC5314, we demonstrated that, indeed, catalase activity of *C. albicans* is critical for increased survival of *S. mutans* against an exogenous H_2_O_2_ challenge when both species are in close proximity. Interestingly, in *C. albicans*, catalase is an ROS-inducible intracellular enzyme that has also been found extracellularly in the cell wall (32). Thus, it appears that *C. albicans* attempts to detoxify ROS derived from the host or other sources before it can damage intracellular components (52 – 54), and thus neighboring ROS-sensitive cells can benefit from these potent detoxification mechanisms.

Clinically, this increased H_2_O_2_ tolerance seen for *S. mutans* when co-cultivated with *C. albicans* can be alarming not only from the perspective that H_2_O_2_ is used as an oral antiseptic but also because H_2_O_2_ produced by peroxigenic bacteria plays an important role in maintaining oral biofilm homeostasis (18). Similar protection of the strictly anaerobic pathogen *Porphyromonas gingivalis* by the catalase-positive *Aggregatibacter actinomycetemcomitans* showed that *A. actinomycetemcomitans’* catalase protects *P. gingivalis* from the oxidative stress of aerobic conditions as well as H_2_O_2_ produced by the peroxigenic commensal *S. sanguinis* (52). Similarly, we showed that *C. albicans*’ catalase protects early *S. mutans-C. albicans* biofilms from interference by sublethal levels of added H_2_O_2_ or the addition of peroxigenic commensal *Streptococcus* A12. The addition of exogenous catalase to single species UA159 and UA159-SC5314Δ*cat1* biofilms alleviated the reduction in biomass following treatment with S*treptococcus* A12, suggesting that catalase at least in part plays a direct role in *S. mutans* oxidative stress protection. Furthermore, we showed that *C. albicans’* catalase prevents *S. mutans* from mounting an oxidative stress response when exposed to sublethal oxidative stress, promoting a favorable environment for *S. mutans* growth and biofilm development. We also observed that when *S. mutans* was in biofilms with SC5314*∆cat1*, an oxidative stress response was still mounted to a lesser extent compared to *S. mutan*s single-species biofilms. Since *C. albicans* possesses six superoxide dismutases(SODs - three extracellular), we speculate that these enzymes could aid in ROS detoxification and provide partial ROS protection to *S. mutans* when co-cultivated with SC5314*∆cat1* and challenged with H_2_O_2_ (53). Studies are underway to determine whether *C. albicans* extracellular SODs also aid in the protection of *S. mutans* against oxidative stress.

While oral infection with either *S. mutans* or *C. albicans* is known as a caries risk factor (4, 54), co-infection with both species is also a risk factor for caries development and reoccurrence (4, 39, 55). Thus, uncovering the major mechanism responsible for the increased oxidative stress tolerance of *S. mutans* in dual-species biofilms with *C. albicans* provides another piece of the puzzle to understand this complex, often synergistic, cross-kingdom relationship. The knowledge that *C. albicans’* catalase protects *S. mutans* in close contact with H_2_O_2_ stress highlights the importance of developing strategies that target the disassociation of these partners in grime for the successful prevention and treatment of dental caries. Recently, Aljaffari and colleagues showed that the use of nystatin, an antifungal rinse, reduced the burden of *S. mutans* in saliva and plaque in patients with thrush, suggesting that approaches limiting colonization by *C. albicans* might help in the prevention and management of caries (56). Thus, when crafting new strategies to effectively combat caries, a multipronged approach including molecules that disrupt the synergism between caries-associated microorganisms, prebiotics that favor antagonistic properties of health-associated microorganisms, and the introduction of beneficial commensals possessing distinct pathways that inhibit different cariogenic traits of caries-associated microorganisms should be considered.

## MATERIALS AND METHODS

### Strains and growth conditions

*Streptococcus mutans* clinical strains SMP1, SMP2, SMP3, SMP4, and SMP5, and the laboratory strain UA159 and its derivative glucosyltransferase B/C mutant (UA159*ΔgtfB/C*) (47) were used in this study. Clinical isolates of *Candida glabrata* CGP5, *Candida tropicalis* CTP4, and *Candida albicans* CAP1, CAP2, and CAP3, as well as the *C. albicans* laboratory strain SC5314 and its derivative catalase mutant SC5314 (SC5314*Δcat1*) were also used. Additionally, the peroxigenic *Streptococcus* A12 was included in biofilm stability assays. Strains were routinely cultured on brain heart infusion (BHI) agar for 48 h at 37°C (5% CO_2_ for streptococci or aerobically for *Candida* species). For all experiments except biofilm stability assays, UA159, UA159*ΔgtfB/C*, SC5314, SC5314*Δcat1,* and clinical strains of *S. mutans* and *Candida* were grown separately in TYE broth containing 1% glucose (w/v) (TYEG). After 18 h incubation at 37°C in 5% CO_2_, these starter cultures were diluted 1:16 in TYEG and incubated until reaching the mid-log growth phase (OD_600 nm_ 0.5 for *S. mutans* and OD_600 nm_ 0.3 for *Candida*). Next, for biofilm inoculations, these cells were harvested by centrifugation (2,147 × *g*, 20 min, 4°C) and resuspended in fresh TYE containing 1% sucrose (TYES) (57) to a final concentration of ~3 × 10^8^ CFU/mL of *S. mutans* (equal TYES volume) and ~3 × 10^6^ CFU/mL for *Candida* (half of the TYES volume).

### Construction of the catalase mutant in *C. albicans*

The catalase (*CAT1*) gene in *C. albicans* was deleted using a CRISPR-based approach, as described (58, 59). Briefly, three separate sets of PCR reactions created linear DNA constructs for (a) codon-optimized Cas9 with the ENO1 promoter, (b) a guide RNA targeting the *CAT1* ORF fused to the Pol III promoter of the SNR52 snoRNA gene, and (c) a repair construct in which the SAT1-FLP construct is amplified using primers that also contain homology to the 5′ and 3′ UTR of *CAT1* (Primers in Table S1) (60). All three PCR products were transformed into *C. albicans* strain SC5314 and selected on media containing nourseothricin. Homozygous deletions were identified by PCR genotyping, and the strains were restored to nourseothricin sensitivity by growth on maltose, which activates the FLP recombinase in the repair construct, leaving the CAT1 ORF replaced with the 34-bp FLP Recognition Target sequence. We generated two independent strains, SC5314∆*cat1* and SC5314∆*cat1#2* that showed the expected peroxide-sensitivity phenotype.

### Biofilm biomass assay

Single- and dual-species biofilms were grown in polystyrene microtiter plates, and wells were coated with filter-sterilized pooled saliva as previously described (57). Saliva was collected from healthy volunteers following informed consent (IRB#201600877). Starter cultures of UA159, UA159*ΔgtfB/C*, SC5314, and SC5314*Δcat1*, prepared as described above, were diluted 1:10 in TYES (~3 × 10^7^ CFU/mL of *S. mutans,* ~3 × 10^5^ CFU/mL of *C. albicans*) to inoculate the wells, followed by incubation (37°C, 5% CO_2_, 48 h; with media replacement at 24 h). Biomass quantification using 0.1% crystal violet staining was done as described elsewhere (57).

To determine whether *C. albicans’* catalase contributes to biofilm stability during exposure to a concentration of H_2_O_2_ that can be normally encountered in the oral cavity (18), early single- and dual-species biofilms of *S. mutans* and *C. albicans* were prepared as above and treated with 0.005% H_2_O_2_ or inoculated with the peroxigenic commensal *Streptococcus* A12 as described in (61) with the exception that both starter cultures and subcultures were initiated in BHI due to the fastidious nature of *Streptococcus* A12. Cultures were diluted 1:100 (~3 × 10^6^ CFU/mL of *S. mutans,* ~3 × 10^4^ CFU/mL of *C. albicans*) in BHI + 1% sucrose (BHIS) to inoculate biofilms. Following biofilm establishment (37°C, 5% CO_2_, 8 h), half of the culture medium was replaced with fresh BHIS, BHIS + 0.01% H_2_O_2,_ or *Streptococcus* A12 culture. *Streptococcus* A12 was previously grown for 18 h in BHI (5% CO_2_, 37°C), diluted to OD_600nm_ = 0.5 ± 0.05 (~3 × 10^6^ CFU/mL), then centrifuged (4,000 rpm, 20 min, 4°C), and resuspended in an equal volume of BHIS. Biofilms were further incubated for 12 h (37°C, 5% CO_2_), followed by crystal violet staining.

### Biofilm oxidative stress survival

Single- and dual-species biofilms were grown on saliva-coated hydroxyapatite (HA) discs as described elsewhere with minor modifications (12). Briefly, HA discs (HiMed Inc., 5 mm diameter × 1.8 mm thick) were saliva-coated, placed in 24-well plates, and inoculated with *S. mutans* (clinical isolates, UA159 or UA159*ΔgtfBC*) and *Candida* (clinical isolates, SC5314 and SC5314*Δcat1*) cultures (~3 × 10^7^ CFU/mL for *S. mutans,* ~3 × 10^5^ CFU/mL for *Candida*). The culture medium was replaced after 24 h with fresh TYES, and biofilms were allowed to grow for a total of 48 h (37°C in 5% CO_2_). Then, biofilms were treated with 0.25% H_2_O_2_ for 0, 30, 60, and 90 min. At each time point, 5 µL of catalase (2,000 U/mL) was added for 2 min. Then, HA discs were dip-washed three times in PBS and placed in a tube with 1 mL PBS, followed by water-bath sonication at medium intensity (10 min) and vortexing (30 s). Serial dilutions were plated on selective media for CFU enumeration of *S. mutans* (Mitis Salivarius Bacitracin agar; MSB) and *Candida* (Sabouraud; SAB).

### Contact-dependence assay

To determine whether the protection conferred by *C. albicans* to *S. mutans* against H_2_O_2_ is contact-dependent, *S. mutans* and *C. albicans* biofilms were grown physically separated in transwell plates (24 mm Costar Transwell plate with 0.4 um pore size). Starter cultures of *S. mutans* UA159 and *C. albicans* (SC5314 and SC5314*Δcat1*) were grown as described in the growth conditions above, then diluted 1:10 in TYEG to inoculate UA159 in the bottom well. SC5314, SC5314*Δcat1*, or TYEG (control) were placed in the insert, and cultures were incubated (12 h, 37°C, 5% CO_2_). Then, the top insert containing *C. albicans* was removed, and the UA159 cultures were treated with 0.2% H_2_O_2_ for 0, 30, 60, and 90 min. An aliquot of the culture was removed at each time, and serial dilutions were plated on BHI agar (48 h, 37°C, 5% CO_2_) for CFU estimation. Control biofilms consisted of co-inoculation with *S. mutans* and *C. albicans* into the bottom well (12 h, 37°C, 5% CO_2_).

To determine whether the supernatant of *C. albicans* cultures pre-exposed for 18 h to 0.01% H_2_O_2_ to induce catalase expression (62) could protect *S. mutans* from H_2_O_2_ killing, 48 h UA159 biofilms were transferred to filter-sterilized supernatants of *C. albicans* SC5314 or fresh TYES, followed by treatment with 0.25% H_2_O_2_ for 0, 30, 60, and 90 min and CFU enumeration.

### Confocal laser scanning microscopy (CLSM)

To gain insight into *C. albicans’* catalase influence on biofilm composition and architecture, UA159, SC5314, and SC5314*Δcat1* cultures were prepared as described above. Briefly, 48 h single- and dual-species biofilms were cultured in TYES on saliva-coated ibidi µ-slide eight chamber coverslips (Cat. No. 80826), with media replacement after 24 h. Biofilms were treated with hydrogen peroxide (0.25% H_2_O_2_ in 0.89% saline solution, 60 min). H_2_O_2_ was neutralized with 5 µL of 2,000 U/mL catalase and rinsed with 0.89% saline solution. Prior to imaging, the biofilms were stained for 30 min in the dark using the Live/Dead bacterial viability kit (Molecular Probes Inc., Eugene, OR, USA) to determine the *S. mutans* viability, whereas *C. albicans* cells were stained with calcofluor white M2R blue-fluorescence fungal surface labeling reagent (Molecular Probes Inc., Eugene, OR, USA). The dyes were excited and detected per the manufacturer’s instructions. The confocal images of biofilms were acquired using a Nikon Ti2 confocal microscope and a Nikon C2plus camera equipped with a Plan Apo λ 60× oil objective with sequential illumination, a z-step of 1 µm, and a 1-s scanning speed. Data represent the average of results obtained from at least four biological replicates (with three images per replicate). The total and individual component biovolume was quantified using the Nis Elements 5.0 Imaging Software (63).

### Oxidative stress gene expression in *S. mutans*

To determine if *C. albicans* protects *S. mutans* from sensing oxidative stress when exposed to a sublethal dose of H_2_O_2_, single-species of *S. mutans* and dual-species (*S. mutans-C. albicans* SC5314, or *S. mutans-C. albicans* SC5314*Δcat1*) biofilms were exposed to 0.005% H_2_O_2_ and expression of *S. mutans’* oxidative stress genes was quantified through qRT-PCR. Briefly, biofilms were grown in six-well plates in TYES (24 h at 37°C, 5% CO_2_) and treated with 0.005% H_2_O_2_ for 5 min, followed by neutralization with 5 µL catalase (2,000 U/mL). After a wash with PBS, biofilms were harvested in RNAprotect (Qiagen), and RNA was isolated by acid-phenol-chloroform extraction, followed by treatment with DNase I (Ambion) (64). The RNA was precipitated and purified with RNeasy Mini Kit (Qiagen) followed by a second on-column DNase treatment (Rnase Free-Dnase, Qiagen) as described elsewhere (35, 65). The NanoDrop One Spectrophotometer (Thermo Fisher Scientific, Waltham, MA) was used to determine RNA concentrations, and samples were run on agarose gels to verify RNA integrity. A high-capacity cDNA reverse transcription kit containing random primers (Applied Biosystems) was used to synthesize cDNA from 1 µg of RNA from each of three independent RNA samples. Quantitative real-time PCRs (qRT-PCRs) were carried out using gene-specific primers (35) and performed in an C1000 Thermal Cycler apparatus (Bio-Rad) (66). Data were normalized by time 0 (untreated) and represented the average and standard deviation of fold change.

### Statistical analysis

All experiments were performed with at least three independent biological replicates. The GraphPad Prism 9.2.0 software (GraphPad Software, La Jolla, CA, USA) was used to analyze the data. For H_2_O_2_ killing, transwell assays, CLSM, and biofilm stability assays, ANOVA followed by Dunn’s or Tukey’s multiple comparison tests were used. For all tests, a *P*-value of <0.05 was considered the threshold for significance.

## SUPPLEMENTAL MATERIAL

Figure S1 msphere.00295-23-s0001.tif Biomass of single and dual species *S. mutans-C. albicans* biofilms is independent of *C. albicans* catalase.

Figure S2 msphere.00295-23-s0002.tif Acute oxidative stress tolerance of *S. mutans* (A) and *C. albicans* (B) in dual- and single-species biofilms with the secondary *C. albicans* catalase mutant.

Figure S3 msphere.00295-23-s0003.tif Representative confocal microscopy images of 48-h-old *S. mutans* and *C. albicans* single-species biofilms.

Figure S4 msphere.00295-23-s0004.tif The effect of *Streptococcus* A12 biofilm biomass reduction of S. mutans and *C. albicans* in dual- and single-species is H_2_O_2_ dependent.

Table S1 msphere.00295-23-s0005.tif List of primers used to generate the *cat1* mutant in *C. albicans*.

ASM does not own the copyrights to Supplemental Material that may be linked to, or accessed through, an article. The authors have granted ASM a non-exclusive, world-wide license to publish the Supplemental Material files. Please contact the corresponding author directly for reuse.
