# Supplementary figures and images for: Catalase produced by Candida albicans protects Streptococcus mutans from H2O2 stress—one more piece in the cross-kingdom synergism puzzle
Source: mSphere. 2023 Aug 21;8(5):e00295-23. doi: 10.1128/msphere.00295-23 (PMC10597455; doi:10.1128/msphere.00295-23)

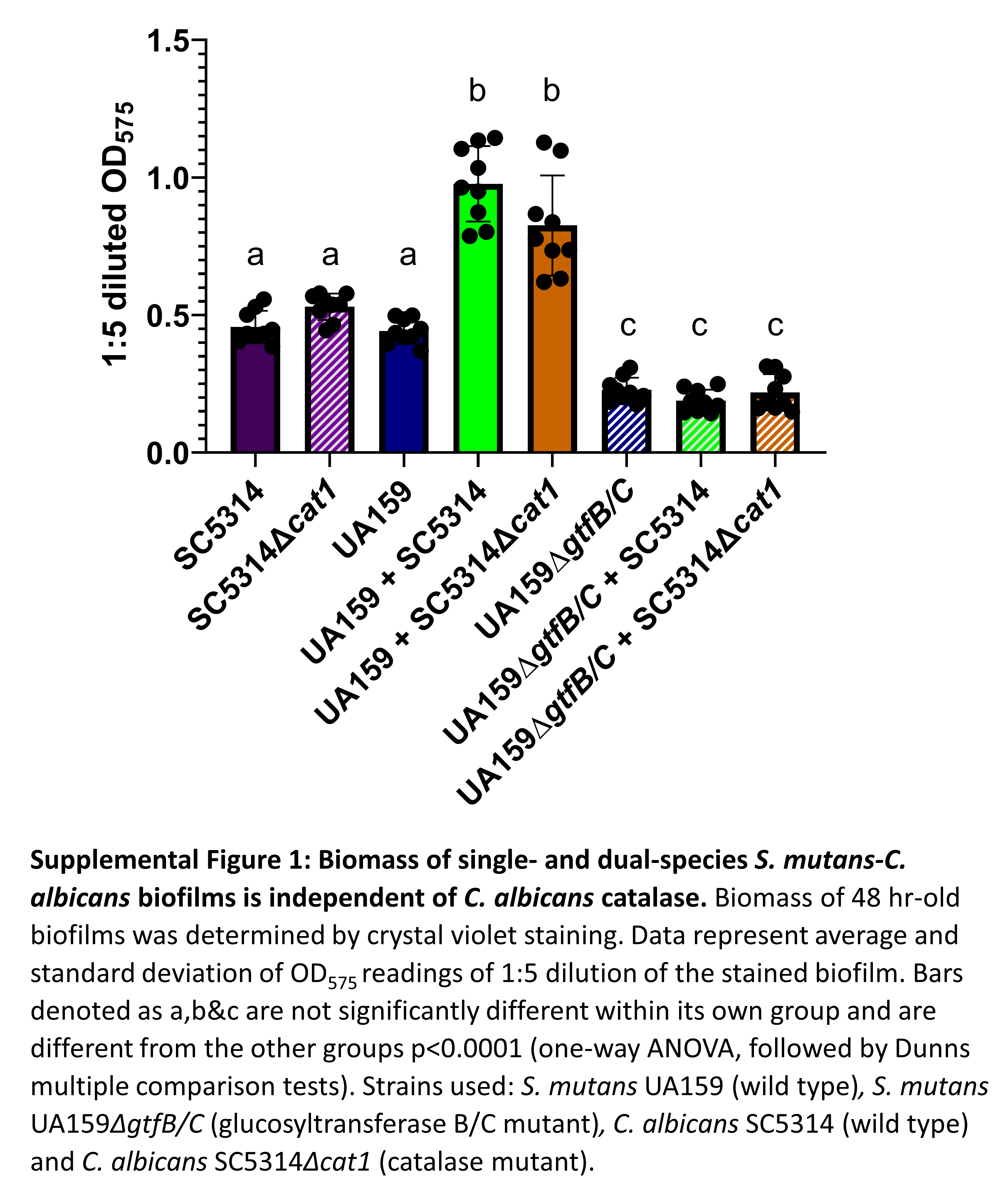

Supplement: Figure S1 — Biomass of single and dual species S. mutans-C. albicans biofilms is independent of C. albicans catalase. [file msphere.00295-23-s0001.tif]

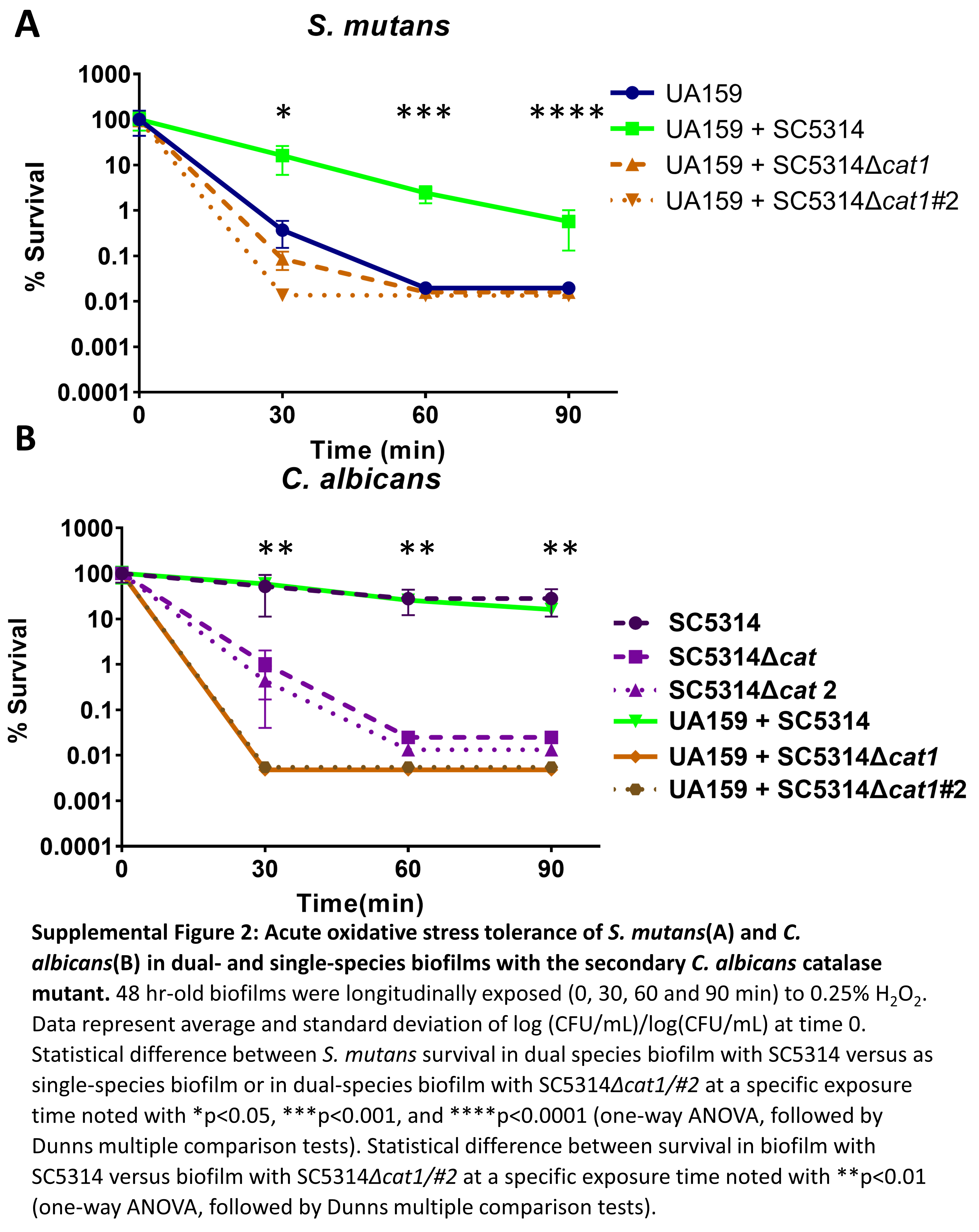

Supplement: Figure S2 — Acute oxidative stress tolerance of S. mutans (A) and C. albicans (B) in dual- and single-species biofilms with the secondary C. albicans catalase mutant. [file msphere.00295-23-s0002.tif]

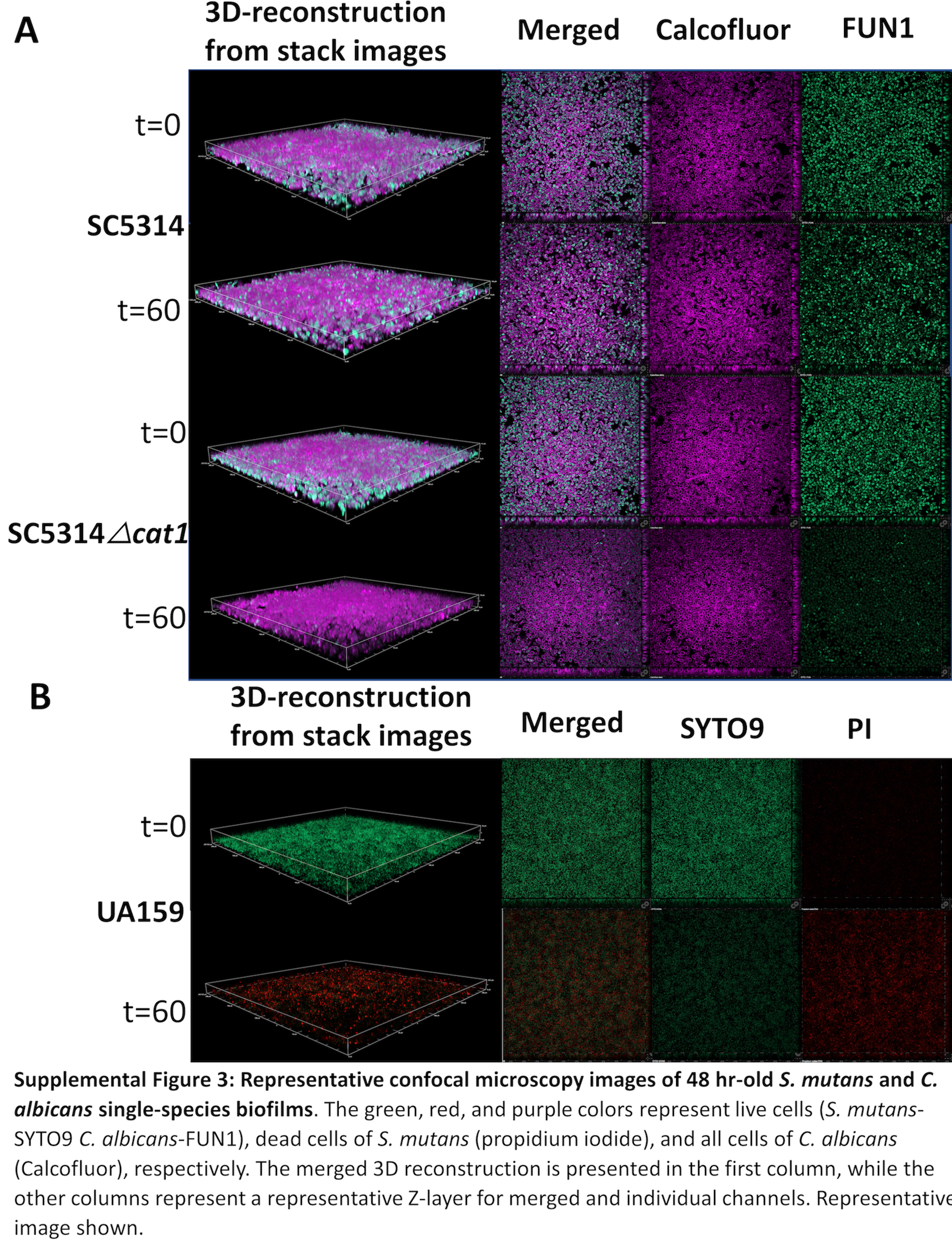

Supplement: Figure S3 — Representative confocal microscopy images of 48-h-old S. mutans and C. albicans single-species biofilms. [file msphere.00295-23-s0003.tif]

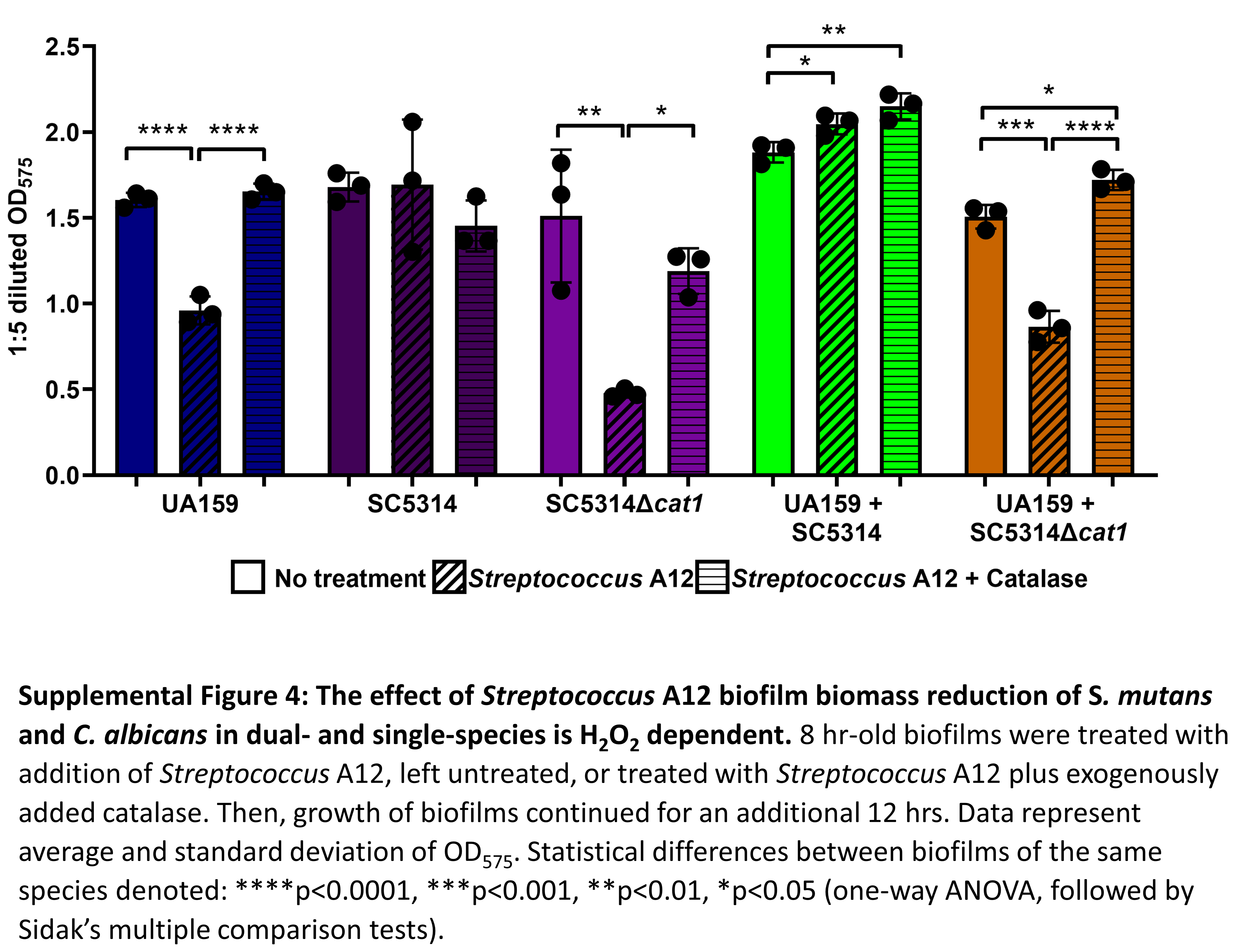

Supplement: Figure S4 — The effect of Streptococcus A12 biofilm biomass reduction of S. mutans and C. albicans in dual- and single-species is H2O2 dependent. [file msphere.00295-23-s0004.tif]

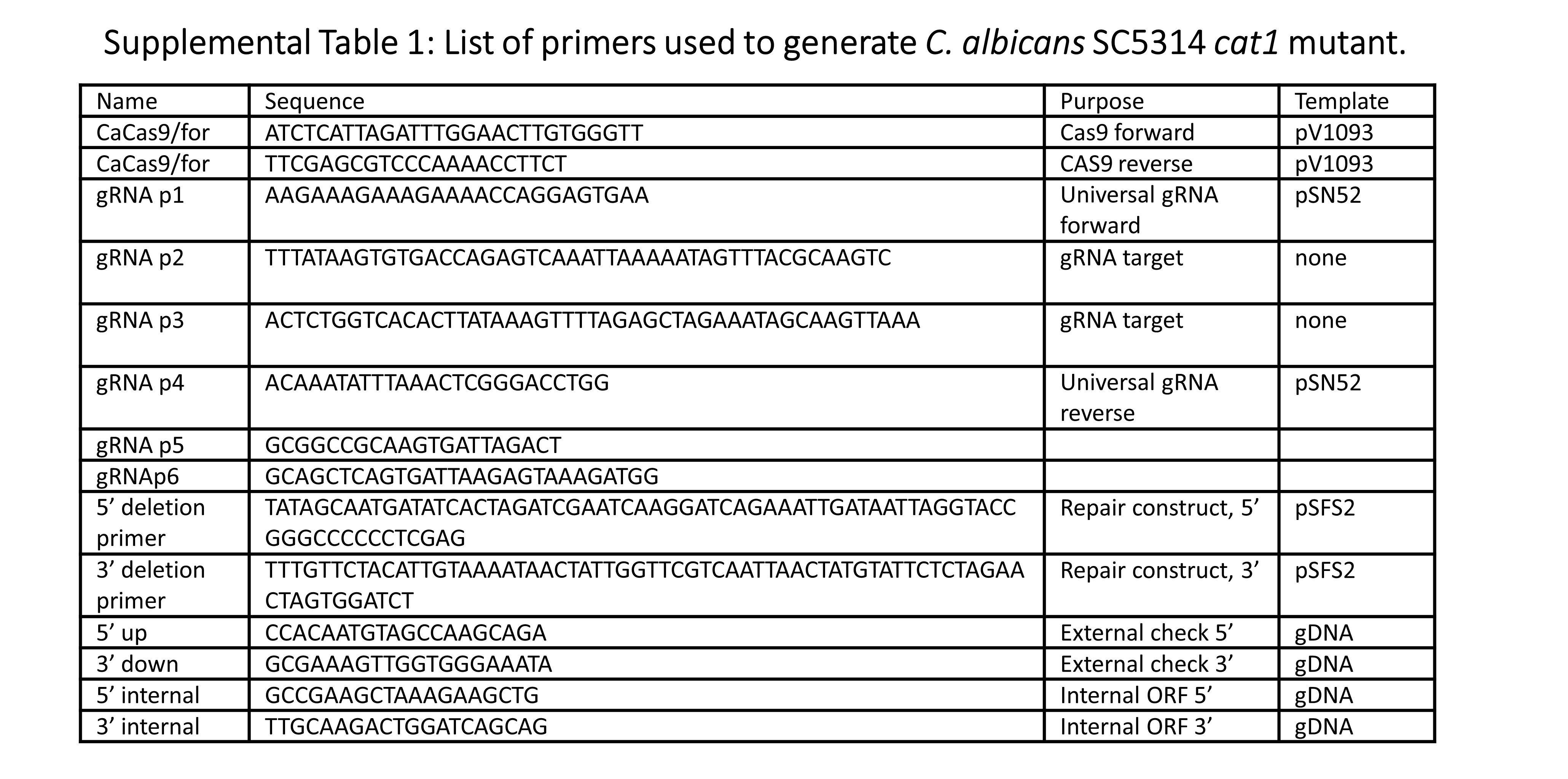

Supplement: Table S1 — List of primers used to generate the cat1 mutant in C. albicans. [file msphere.00295-23-s0005.tif]
